# Meta-analysis of gemcitabine in brief versus prolonged low-dose infusion for advanced non-small cell lung cancer

**DOI:** 10.1371/journal.pone.0193814

**Published:** 2018-03-21

**Authors:** Zhao Dehua, Chu Mingming, Wang Jisheng

**Affiliations:** 1 Dept. of Clinical Pharmacy, The Third Hospital of Mianyang, Mianyang, Sichuan, China; 2 Dept. of Clinical Pharmacy, The Second Affiliated Hospital of Third Military Medical University, Chongqing, China; Ottawa Hospital Research Institute, CANADA

## Abstract

**Objective:**

To evaluate the efficacy and safety of gemcitabine (GEM) at 30 min standard-dose infusion (30 min-SDI) compared with prolonged low-dose infusion (P-LDI) in patients with advanced non-small-cell lung cancer (NSCLC).

**Methods:**

Electronic databases including Pubmed, EMbase, Cochrane Library, CNKI, CBM, and VIP were searched using keywords “GEM”, “P-LDI”, and “NSCLC”. Review Manager 5.3 was used to perform the meta-analysis. Primary endpoints were overall response rate (ORR) and 1-year survival rate (1-year SR). Secondary endpoints were grade 3/4 hematotoxicity and nausea/vomiting. In association. GRADE quality of evidence system was used to assess the results of meta-analysis.

**Results:**

Six randomized controlled trials (RCTs) with a total of 637 patients were included and no statistical heterogeneity was found among the studies. The results showed that P-LDI was superior in ORR (RD = 0.09, 95% CI: 0.02 to 0.16, P = 0.02), but had a similar 1-year SR (RD = 0.05, 95% CI: -0.02 to 0.12, P = 0.18) as compared with 30 min-SDI. For grade 3/4 adverse events, there was no significant difference in anemia (RD = 0.02, 95% CI: -0.01 to 0.04, P = 0.27) and nausea/vomiting (RD = 0.01, 95% CI: -0.04 to 0.06, P = 0.64) between the two treatments. However, patients with P-LDI experienced less leukopenia (RD = -0.08, 95% CI: -0.15 to -0.01, P = 0.03) and thrombocytopenia ((RD = -0.05, 95% CI: -0.09 to –0.01, P = 0.006). The GRADE profile showed that the included RCTs had low quality of evidences.

**Conclusion:**

P-LDI was superior in terms of ORR, experienced less grade 3/4 thrombocytopenia and leukopenia compared with 30 min-SDI, and could be a viable treatment option for advanced NSCLC. However, the results need to be further verified by high quality trials and large samples owing to the low quality of evidences.

## Introduction

Lung cancer is the most common cause of cancer-related deaths, and NSCLC accounts for most of these cases [1] (85% to 90%). Many patients with NSCLC have locally advanced or metastatic disease at the time of diagnosis, and the overall survival is poor [2]. For patients with targetable mutations (such as EGFR and ALK), tyrosine kinase inhibitor (TKI) is considered as the first-line treatment regimens. On the other hand, for patients with no targetable mutations, platinum drugs combined with the third-generation antineoplastic agents, such as paclitaxel, docetaxel, GEM, vinorelbine and pemetrexed, is considered the standard of care for patients with unresectable or advanced NSCLC [3–5].

GEM is a pyrimidine antimetabolite, structurally related to cytosine arabinoside (Ara-C) [6], and is effective in treating a wide range of solid tumors. Currently, GEM combined with platinum is one of the standard chemotherapy regimens for patients with advanced NSCLC [4,5]. In clinical practice, GEM at 1000 mg/m^2^ is given as a 30-min infusion. Another dose schedule is prolonged infusion of GEM at a fixed dose rate of 10 mg/m^2^/minute, and both of these dose schedules have been demonstrated to be effective and tolerable. However, several phase I and phase II clinical trials [7–10] have shown that GEM with P-LDI has significant antitumor activity and fewer side effects for patients with advanced NSCLC.

Due to the small sample size of each clinical trials, it is not clear that whether P-LDI is superior to 30 min-SDI for advanced NSCLC. Therefore, a meta-analysis was performed to compare the efficacy and safety of P-LDI with 30 min SDI for the treatment of advanced NSCLC.

## Materials and methods

### Literature search strategy

Electronic databases including Pubmed, EMbase,Cochrane Library, CNKI, CBM, VIP were queried, and the most recent search was performed on January 3, 2017. The search was limited to articles published in English and Chinese. Keywords included “gemcitabine”, “GEM”, “prolonged low-dose infusion”, “prolonged infusion”, “long infusion”, “low dose”, “30-min infusion”, “standard dose”, ‘‘non-small-cell lung cancer”, and ‘‘NSCLC”. The references from the included studies and the websites of clinical trials was also examined for additional eligible publications.

### Inclusion criteria

The inclusion criteria were as follows: RCTs with full articles; patients eligible for the trial had cytologically confirmed inoperable or unresectable NSCLC of stageⅠ–Ⅳ; the follow-up time was more than 1 years; studies comparing GEM at P-LDI with 30 min-SDI; endpoints of ORR (PR+CR); 1-year SR; and hematotoxicity and non-hematotoxicity was reported. Response was assessed by using the response evaluation criteria in solid tumors (RECIST)[11], and National Cancer Institute Common Toxicity Criteria (CTC) version 2.0 were used for grading the toxicity[12]. Two investigators selected the eligible trials based on the inclusion criteria independently. Disagreement was addressed by discussion until consensus was achieved.

### Data abstraction

Two investigators extracted data from eligible studies independently, and the items extracted from each study included first author, publication date, journal, intervention group, control group, chemotherapy regimens, number of patients, age, percentage male, ORR, overall survival (OS), progression free survival (PFS), 1-year SR, hematotoxicity, and non-hematotoxicity. We contacted the authors of the primary studies for missing data. If we were unable to contact the authors, we excluded the study.

### Quality assessment

Two investigators used the risk of bias tool (Cochrane Handbook V5.1.0) to assess the quality of trials independently. Sequence generation, allocation concealment, blinding, incomplete data, selective reporting and other sources of bias were assessed. Disagreements between the two investigators were resolved by discussion with a third investigator.

### Statistical analysis

Two investigators used Review Manager 5.3 to perform the statistical analyses. A fixed-effect model was used to calculate risk difference (RD) for ORR, 1-year SR, and side effects, together with a 95% confidence interval (CI) for dichotomous results. OS and PFS were not included because of insufficient data. A RD>0 indicates that P-LDI is associated with a higher ORR, 1-year SR, and more toxicities than 30 min-SDI. The presence of statistical heterogeneity between the studies was assessed by I^2^ statistic using Q statistic. A P≥0.05 or I^2^≤50% indicated that trials are without heterogeneity, and a fixed-effect model was used to perform the meta-analysis. A P<0.05 or I^2^>50% led us to consider a random-effect model to perform the meta-analysis. Publication bias was assessed by the construction of funnel plots.

### Quality evaluation of evidence

GRADE pro 3.2 Software was used to classify the quality of evidence. All of the included studies were RCTs, and the RCT was set as the highest level of evidence. Five factors could reduce the quality of evidence, including risk of bias, inconsistency, indirectness, imprecision and publication bias.

## Results

### Eligible studies

**Fig 1:**
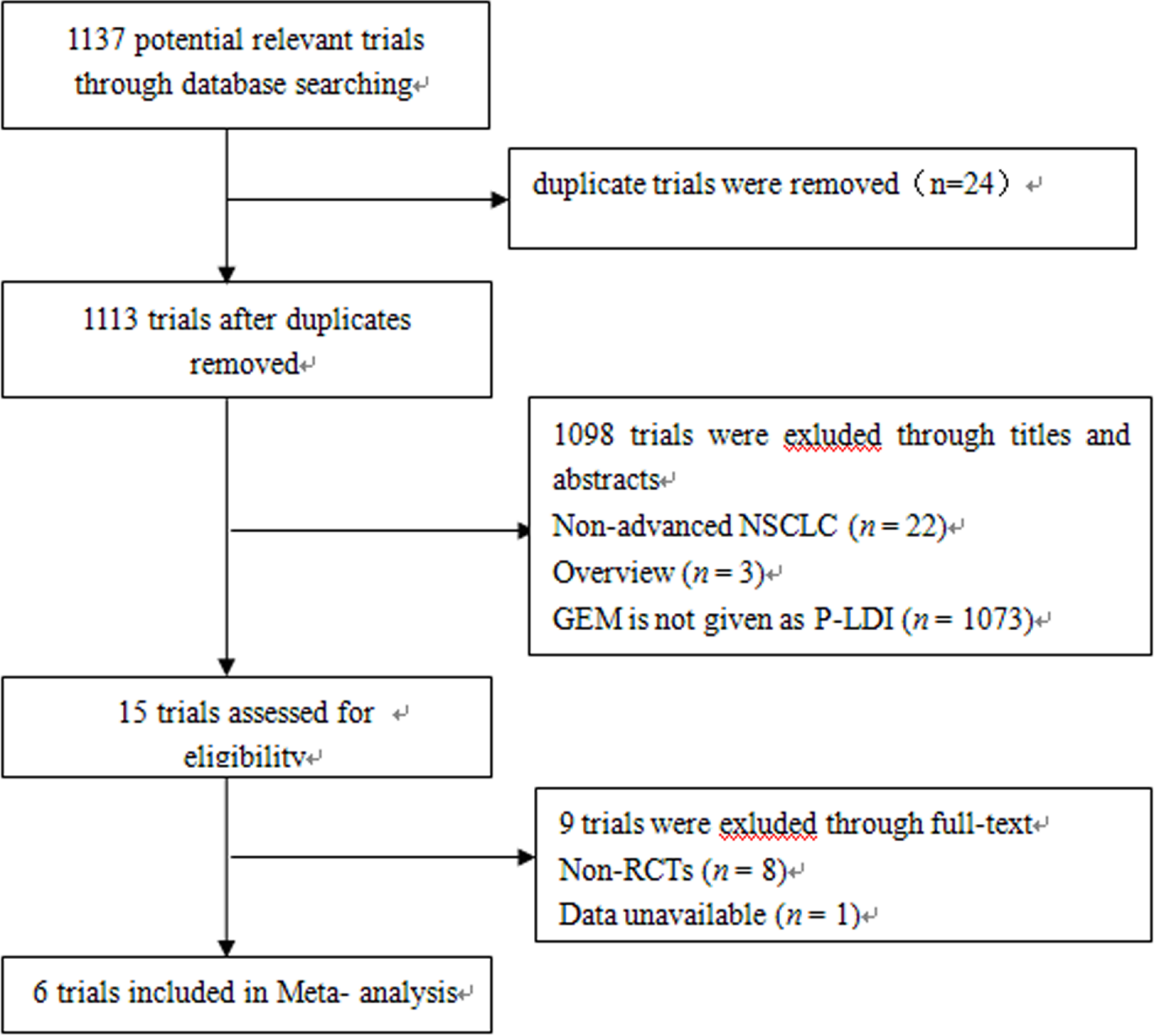
Flowchart of included and excluded trials.

A total of 1137 articles were identified by the initial search strategy. After examining the titles and full-text, six identified RCTs [7–9,13–15] were selected for the meta-analysis (Fig 1). Nine trials were excluded because they were not randomized [6,10,16–21] or because the data was unavailable[22]. The characteristics of the eligible studies are summarized in Table 1.

**Table 1:** Characteristics of eligible trials.

Trials	No.	Male (%)	Age	Stage (No.)	Chemotherapy regimens	Line	PS	Radiation given
Beniwal SK, 2012[13]	30	86.6	53.3 35–65	Ⅲ_B_ /Ⅳ 17/13	GEM (1000 mg/m^2^ in 30 min d1, d8) +CBP (AUC 5 d1). 21 d cycle, 4–6cycles.	First-line	0~2	No
	30	93	54.5 40–70	Ⅲ_B_ /Ⅳ 15/15	GEM (350 mg/m^2^ in 6 h d1, d8) +CBP (AUC 5 d1). 21 d cycle, 4–6 cycles.	First-line	0~2	No
Vrankar M, 2014[7]	52	75	58 42–72	Ⅰ~Ⅱ/Ⅲ_A_ /Ⅲ_B_ 3/19/30	GEM (1250 mg/m^2^ in 30 min d1, d8) +DDP (75 mg/m^2^ d2). 21 d cycle, 3 cycles. Followed with radiotherapy concurrent with DDP + VP16	First-line	0~1	After Chemotherapy
	54	81.5	57 30~77	Ⅰ~Ⅱ/Ⅲ_A_ /Ⅲ_B_ 2/31/21	GEM (250 mg/m^2^ in 6 h d1, d8) +DDP (75 mg/m^2^ d2). 21 d cycle, 3 cycles. Followed with radiotherapy concurrent with DDP + VP16	First-line	0~1	After Chemotherapy
Zwitter M, 2009[8]	125	76	58 41–77	Ⅲ_B_ /Ⅳ 9/116	GEM (1250 mg/m^2^ in 30 min d1, d8) +DDP (75 mg/m^2^ d2). 22 d cycle, 4 cycles. continued with two additional cycles of GEM as monotherapy.	Unclear	0~2	No
	124	75	59 40–79	Ⅲ_B_ /Ⅳ 11/113	GEM (250 mg/m^2^ in 6 h d1, d8) +DDP (75 mg/m^2^ d2). 22 d cycle, 4 cycles. continued with two additional cycles of GEM as monotherapy.	Unclear	0~2	No
Zwitter M, 2010[9]	57	80.7	66 41–81	Ⅲ_B_ /Ⅳ 2/55	GEM (1250 mg/m^2^ in 30 min d1, d8) +DDP (60 mg/m^2^ d2). 21 d cycle, 2–6 cycles.	First-line	2~3	No
	55	67.3	65 49–80	Ⅲ_B_ /Ⅳ 3/52	GEM (200 mg/m^2^ in 6 h d1, d8) +DDP (60 mg/m^2^ d2). 21 days cycle, 2~6cycles.	First-line	2~3	No
Shang, ZT, 2010[14]	30	21	58 30–64	Ⅲ_B_ /Ⅳ 19/11	GEM (1000 mg/m^2^ in 30 min d1, d8) +DDP (75 mg/m^2^ d1). 21 days cycle, 2~6 cycles.	First-line	0~2	No
	30	20	52 32–75	Ⅲ_B_ /Ⅳ 20/10	GEM (250 mg/m^2^ in 6 h d1, d8) + DDP (75 mg/m^2^ d1). 21 d cycle, 2–6 cycles.	First-line	0~2	No
Xiong JP, 2005[15]	25	16	52 32–68	Ⅲ_B_ /Ⅳ 9/16	GEM (1000 mg/m^2^ in 30 min d1, d8) +DDP (75 mg/m^2^ d1). 21 d cycle, 4 cycles.	First-line	0~2	No
	25	15	56 28–70	Ⅲ_B_ /Ⅳ 8/17	GEM (250 mg/m^2^ in 6 h d1, d8) +DDP (75 mg/m^2^ d1). 21 d cycle, 4 cycles.	First-line	0~2	No

CBP: Carboplatin; DDP: Cisplatin; etoposide: VP16

### Quality and publication bias of included trials

**Fig 2:**
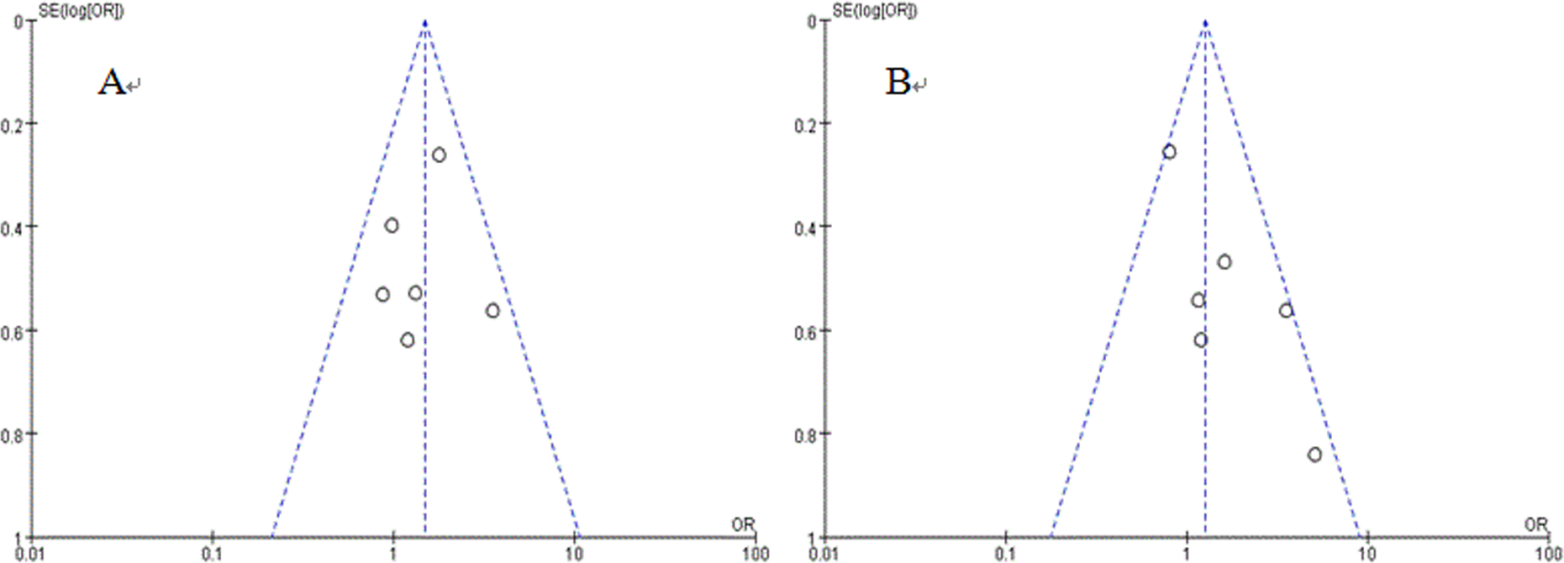
Funnel plots of meta-analysis. A: funnel plots for ORR; B: funnel plots for 1-year SR.

Although participants were randomized into different treatment arms in each trial, there were only two trials presented the detail of sequence generation and blinding, and none of them presented details of allocation concealment, selective reporting, or other sources of bias (Table 2). In summary, the risk of bias and the methodology quality of the included trials were acceptable, and no significant publication bias was detected by using funnel plots (Fig 2).

**Table 2:** Quality evaluation of included trials.

Included trials	Sequence generation	Allocation concealment	Blinding	Incomplete data	Selective reporting	Other sources of bias
Beniwal SK, 2012[11]	Unclear	Unclear	Unclear	No	Unclear	Unclear
Vrankar M, 2014[7]	Unclear	Unclear	Unclear	Yes	Unclear	Unclear
Zwitter M, 2009[8]	Computer-generated sequence of random numbers	Unclear	Single- blind	Yes	Unclear	Unclear
Zwitter M, 2010[9]	Computer-generated sequence of random numbers	Unclear	Single- blind	Yes	Unclear	Unclear
Shang ZT, 2010[13]	Unclear	Unclear	Unclear	No	Unclear	Unclear
Xiong JP, 2005[14]	Unclear	Unclear	Unclear	No	Unclear	Unclear

### Overall response rate (ORR)

**Fig 3:**
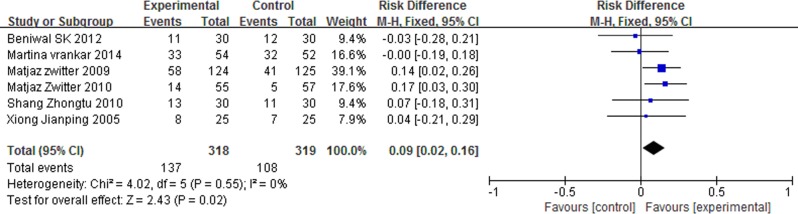
Forest plot of meta-analysis for ORR.

The ORR was defined as the patients who achieved a complete remission (CR) or partial remission (PR). No statistical heterogeneity between studies was found (I^2^ = 0%, P = 0.55). We used a fixed-effect model for meta-analysis, and the results indicated that P-LDI was superior in ORR (RD = 0.09, 95% CI: 0.02 to 0.16, P = 0.02) as compared with 30 min-SDI (Fig 3).

### 1-year survival rate (1-year SR)

**Fig 4:**
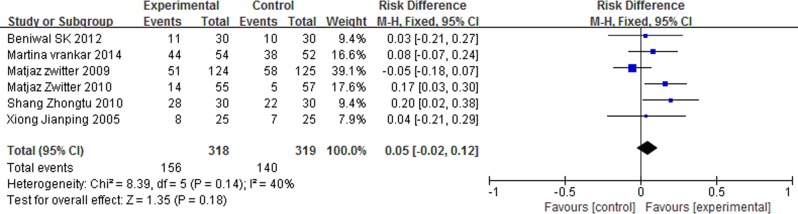
Forest plot of meta-analysis for 1-year SR.

No statistical heterogeneity between studies was found (I^2^ = 40%, P = 0.14), and we used a fixed-effect model. Meta-analysis results indicated that P-LDI had a similar 1-year SR (RD = 0.05, 95% CI: -0.02 to 0.12, P = 0.18) compared with 30 min-SDI (Fig 4). This indicated that there was no statistical difference of 1-year SR between the two arms.

### Subgroup analysis

**Fig 5:**
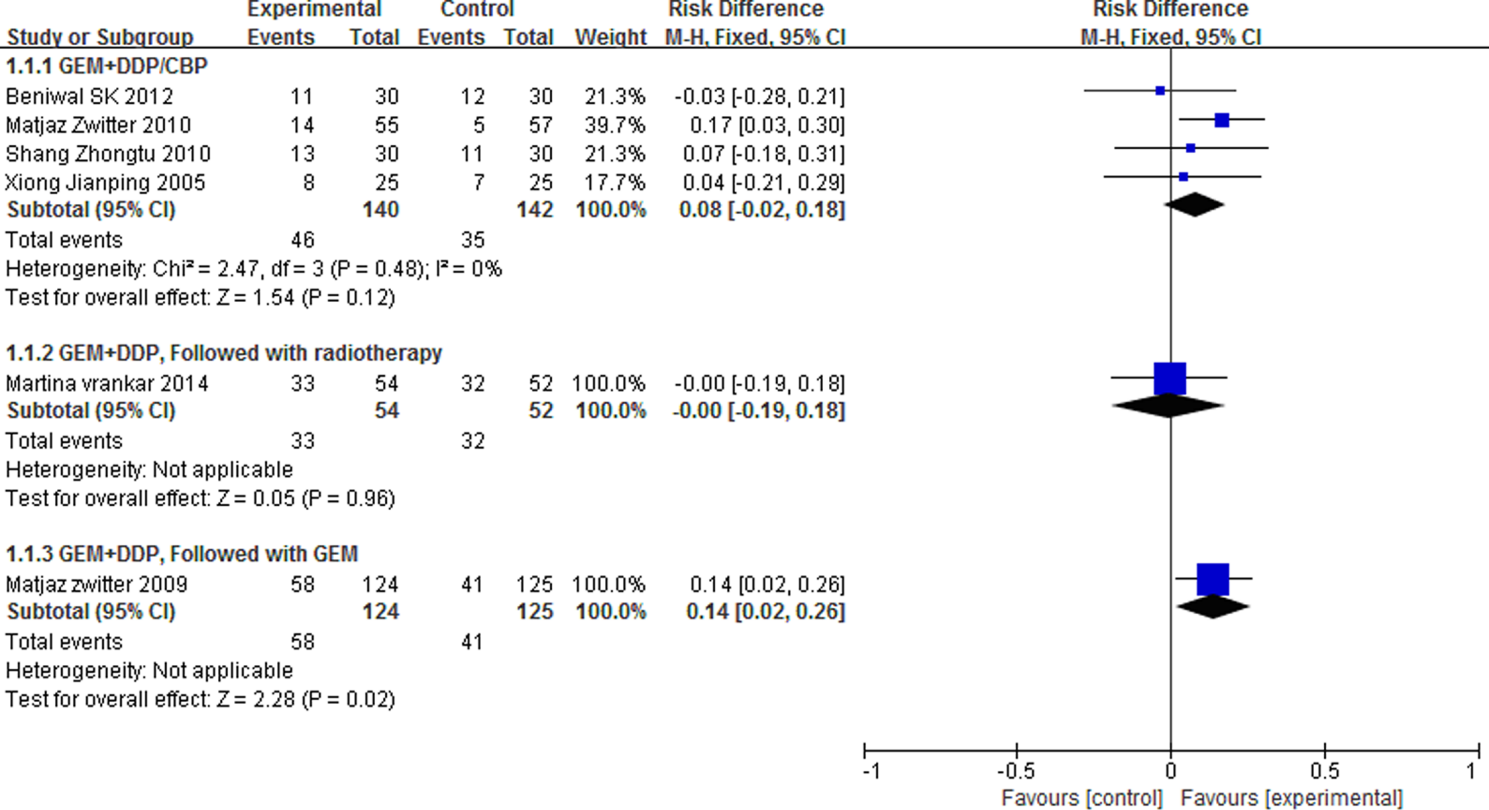
Forest plot of subgroup analysis for ORR.

**Fig 6:**
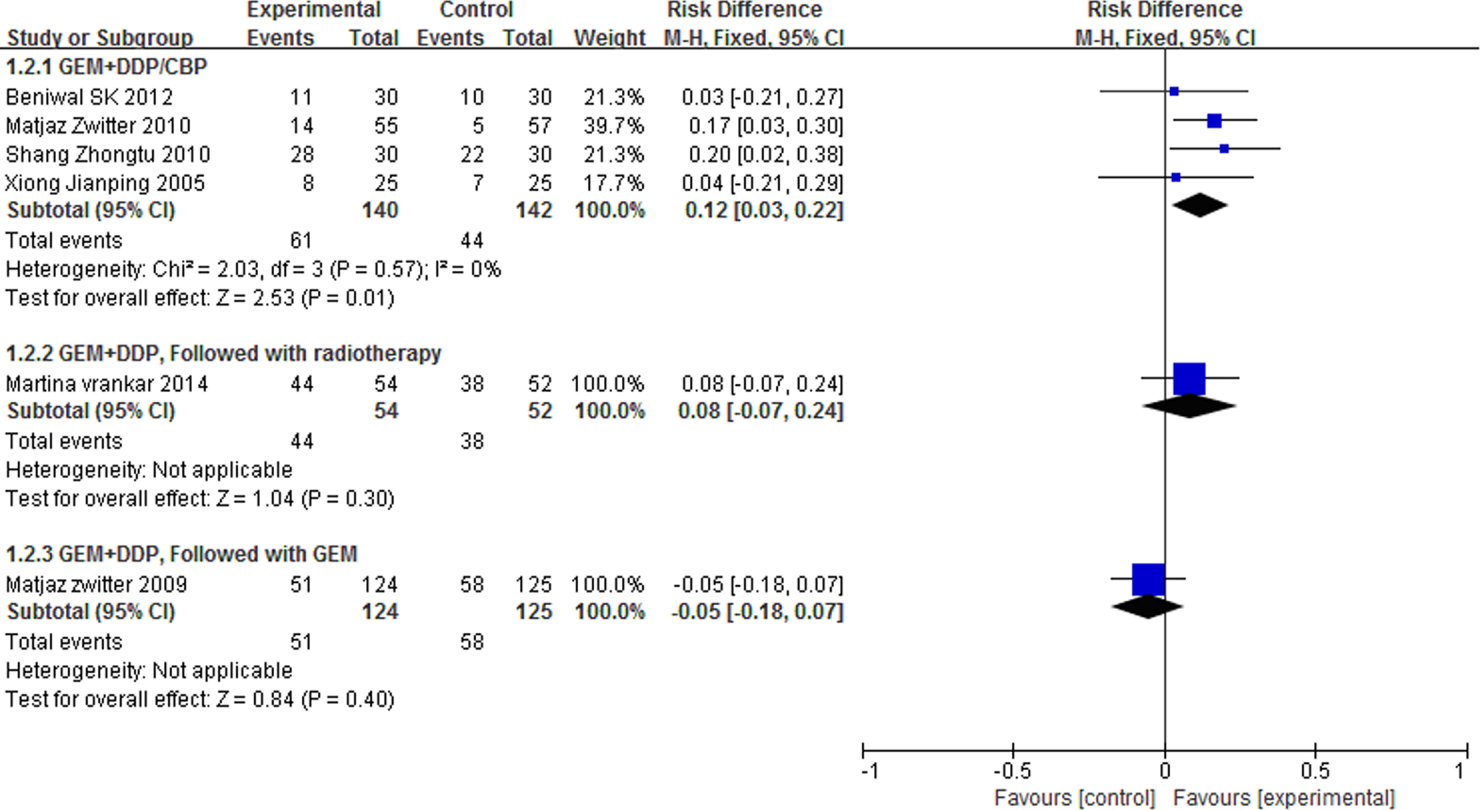
Forest plot of subgroup analysis for 1-year SR.

The were three different schedules for the treatment of advanced NSCLC, including GEM combined with DDP/CBP, GEM combined with DDP and followed with radiotherapy, GEM combined with DDP and followed GEM. So we did a subgroup analysis, and the subgroup analysis showed that P-LDI was superior in ORR as compared with 30 min-SDI for patients who accepted GEM combined with DDP and followed GEM (Fig 5). On the other hand, P-LDI was superior in 1-year SD as compared with 30 min-SDI for patients who accepted GEM combined with DDP/CBP (Fig 6).

### Grade 3/4 adverse events

**Fig 7:**
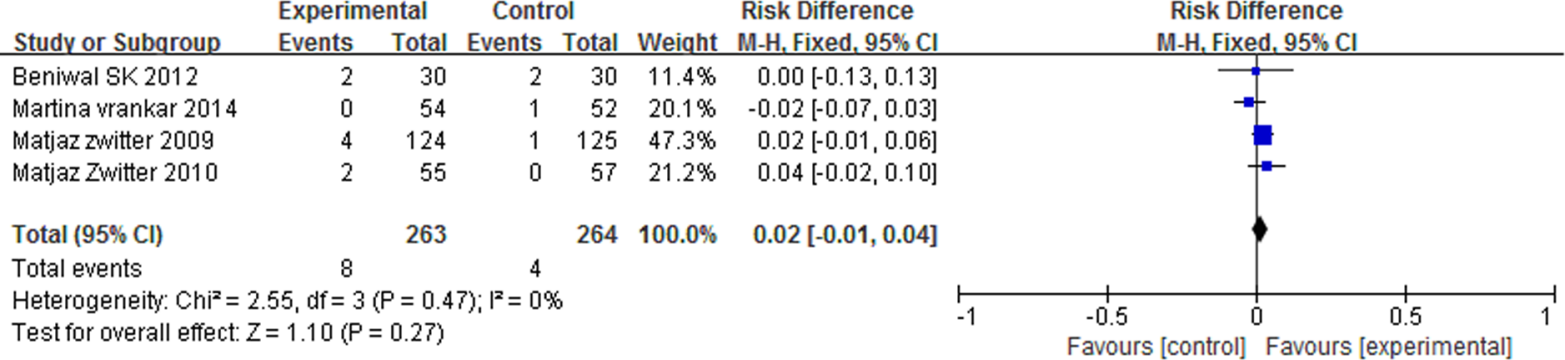
Forest plot of meta-analysis for anemia (grade 3/4).

**Fig 8:**
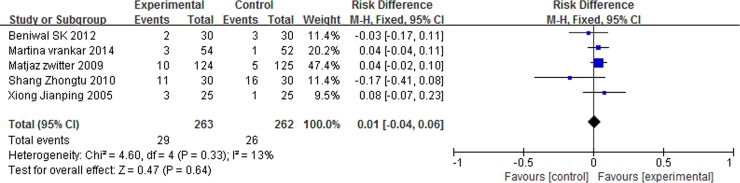
Forest plot of meta-analysis for nausea/vomiting (grade 3/4).

**Fig 9:**
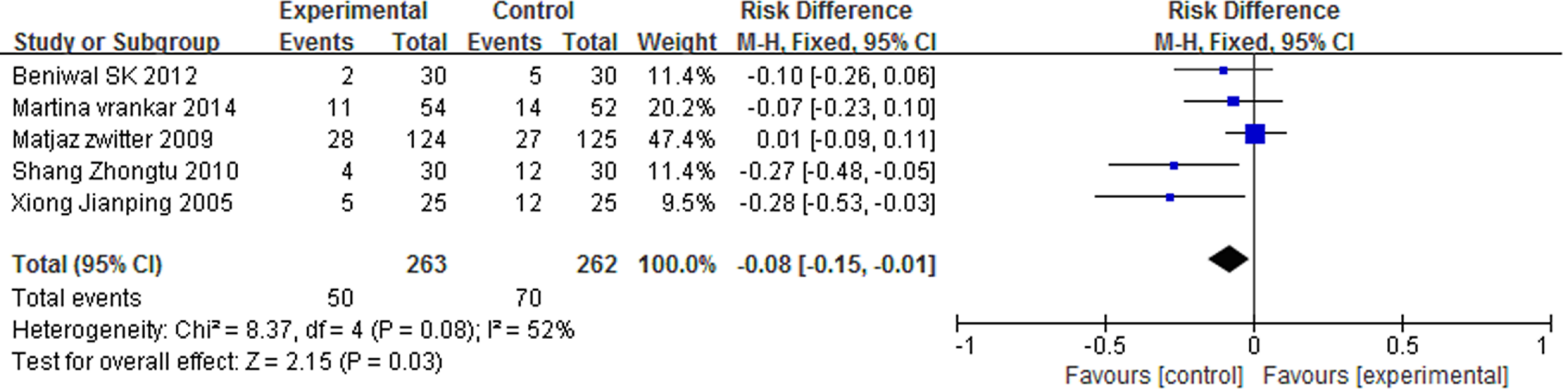
Forest plot of meta-analysis for leukopenia (grade 3/4).

**Fig 10:**
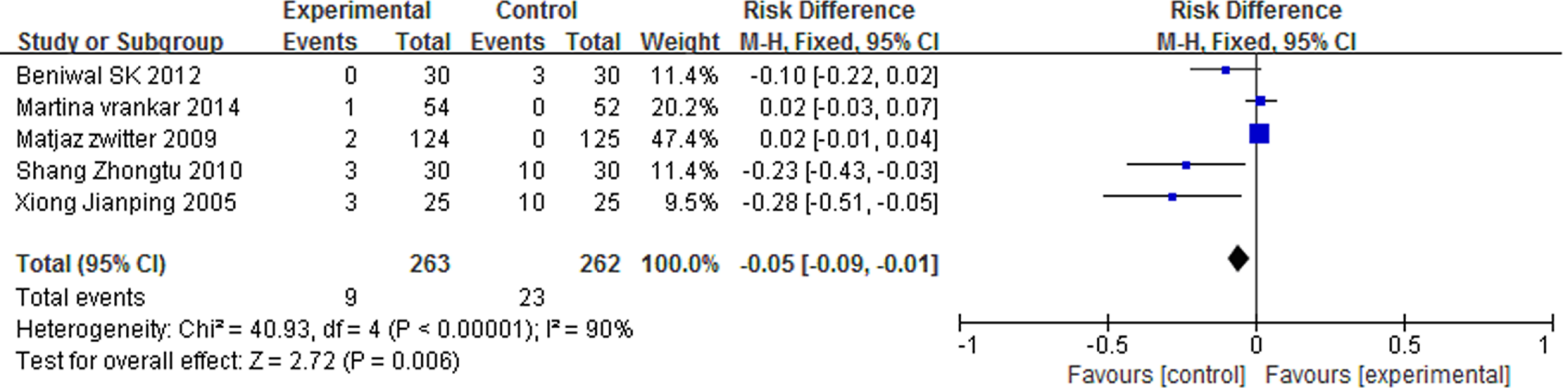
Forest plot of meta-analysis for thrombocytopenia (grade 3/4).

There was no significant differences in anemia (RD = 0.02, 95% CI: -0.01 to 0.04, P = 0.27) and nausea/vomiting (RD = 0.01, 95% CI: -0.04 to 0.06, P = 0.64) between the two arms. However, patients with P-LDI experienced less leucopenia (RD = -0.08, 95% CI: -0.15 to -0.01, P = 0.03) and thrombocytopenia (RD = -0.05, 95% CI: -0.09 to -0.01, P = 0.006) than did patients with 30 min-SDI. (Figs 7 and 8 and 9 and 10).

### Quality evaluation of evidence

**Fig 11:**
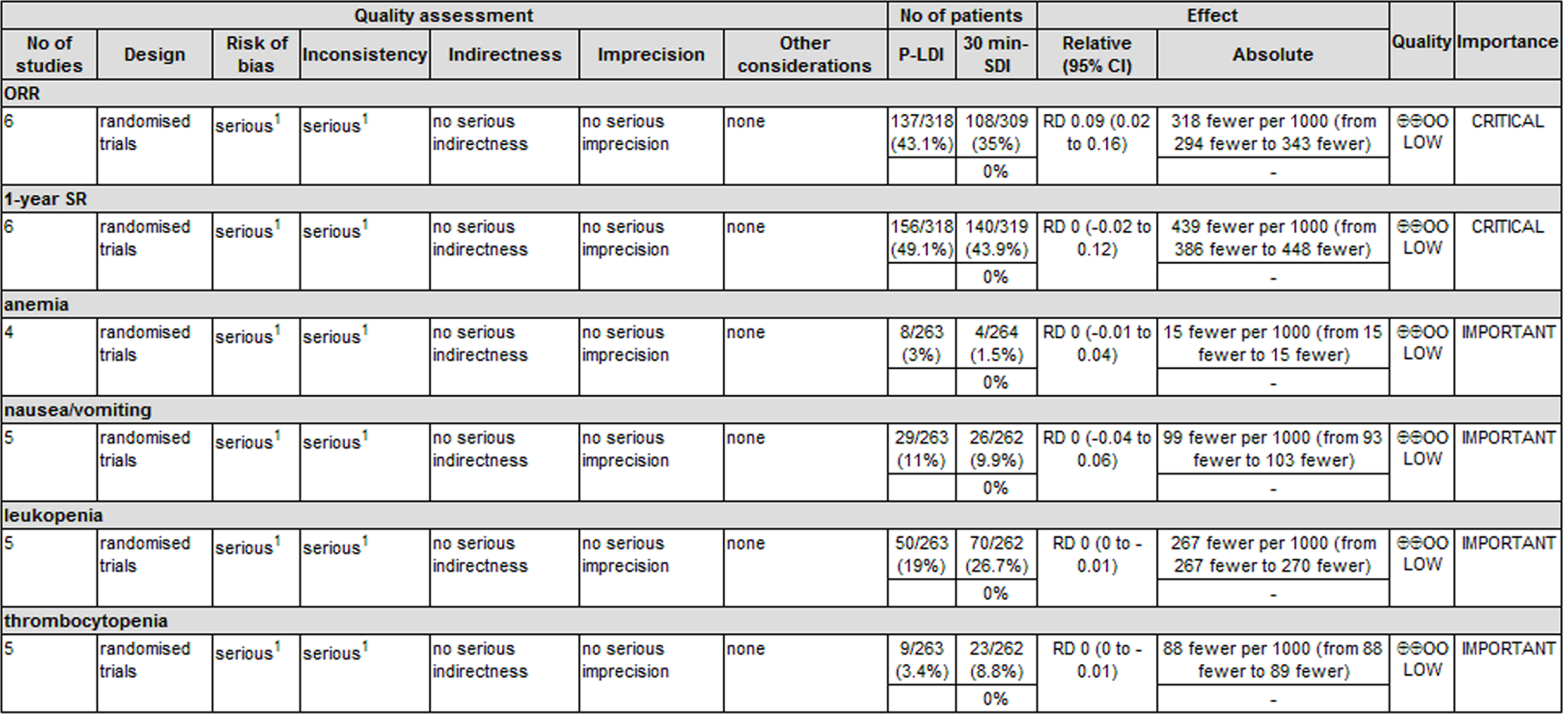
GRADE system for grading the quality of evidence.

When used GRADE profiler software to assess the quality of evidence. According to the GRADE system, it was clearly that all of the outcomes were low in the GRADE system for grading evidence (Fig 11), indicated that the results need to be further verified by high quality trials and large samples.

## Discussion

GEM combined with platinum has been proven to be effective and well tolerated for patients with advanced NSCLC [2]. In several large phase Ⅲ trials [1–3], the ORR ranged from 22 to 40.6%, PFS from 4.2 to 9.8 months, OS from 8.1 to 9.8 months, and 1-year SR from 32 to 39% [2,3].

GEM is transported across the plasma membrane by specific nucleoside transporters and phosphorylated to the triphosphate (dFdCTP) by deoxycytidine kinase (DK) [23]. However, the DK is saturated at concentration of 10–20 μmol/L of GEM, and there is no linear dose-activity relationship between the dFdCTP and the AUC of GEM [24,25]. GEM is usually administered as a 30 min infusion of 1000–1250 mg/m^2^ on days 1, 8, 15, ever 28 d afterwards, and is effective and well tolerated for patients with advanced NSCLC [2]. However, the plasma concentration following 30 min infusion of 1000 mg/m^2^ often exceeds the saturation concentration of DK [23,24]. Thus, by prolonging the infusion time, the plasma concentration of dFdCTP may be increased to achieve better efficiency.

There are three types of infusion for the administration of GEM, including 30 min-SDI, fixed-dose rate (FDR) of 10 mg/m^2^/min infusion, and P-LDI. The 30 min infusion of GEM is the standard regimen. However, some studies [26] have investigated the feasibility and efficacy of FDR in the treatment of NSCLC, and controversial conclusions have been drawn from these trials. A meta-analysis of 6 RCTs [21] demonstrated that FDR of GEM had an equal ORR and 1-year SR as 30 min infusion in patients with advanced NSCLC. Otherwise, FDR was associated with more grade 3/4 hematotoxicity and non-hematotoxicity than 30 min-SDI was.

Another type of infusion is P-LDI, and several clinical trials [7–9] were established to evaluate the efficacy and safety of GEM at 30 min-SDI compared with P-LDI in patients with advanced NSCLC. In a phase I–II trial, GEM with a 6 h infusion in combination with cisplatin was used to the treat advanced NSCLC [10]. During the phase I trial, the dose of GEM ranged from 130 to 250 mg/m^2^, and there was no dose–response relationship in this range. In a phase II trial, the remaining patients received GEM at 250 mg/m^2^ in a 6-h infusion, and the ORR, PFS, OS and 1-year SR were 46%, 6 months, 9.5 months and 40%, respectively [10]. Matjaz Zwitter [9] presented a phase II randomized clinical trial of two schedules of chemotherapy for patients with NSCLC. The response rate was 26.9% and 9.4%, the median PFS was 3.8 and 5.6 months, the median OS was 4.3 and 6.8 months for 30 min-SDI and P-LDI, respectively (*P*<0.05). Another study from Beniwal SK [13] reported that GEM (P-LDI) in combination with carboplatin had an equal activity and low toxicity as compared with 30 min-SDI. In order to evaluate the efficacy and safety of GEM at 30 min-SDI compared with P-LDI in patients with advanced NSCLC, a meta-analysis was performed.

Limitations of this meta-analysis should also be acknowledged. At first, we excluded non-English articles, and most studies included in this meta-analysis included a small size, thus this may lead to a small study effect. Secondly, due to insufficient data of OS and PFS, we did not pool the survival data of OS or PFS. Instead, we utilized other survival metrics, the 1-year SR, to address this limitation. Thirdly, there is no significant difference in 1-year SR, which may be caused by the small number of original studies. Therefore, more studies with large sample sizes are required to answer this question.

## Conclusion

Compared with 30 min-SDI, GEM with P-LDI was superior in ORR and resulted in less grade 3/4 thrombocytopenia and leukopenia in patients with advanced NSCLC. Thus, GEM with P-LDI is a viable treatment option for patients with advanced NSCLC. However, the results need to be further verified by high quality trials and large samples owing to the limited number of RCTs and the poor quality among the included studies.

## Supporting information

S1 File The PRISMA 2009 checklist. (DOC)

S2 File Search strategy. (DOCX)
