# Supplementary material for: Meta-analysis of gemcitabine in brief versus prolonged low-dose infusion for advanced non-small cell lung cancer
Source: PLoS One. 2018 Mar 21;13(3):e0193814. doi: 10.1371/journal.pone.0193814 (PMC5862432; doi:10.1371/journal.pone.0193814)
Supplement: S2 File — (DOCX) [file pone.0193814.s002.docx]

**search strategy**

# 1 Gemcitabine [Tile/Abstract]

#2 non-small-cell lung cancer or NSCLC [Tile/Abstract]

# 3 low-dose [Tile/Abstract]

#4 standard dose [Tile/Abstract]

#5 prolonged infusion or long infusion [Tile/Abstract]

#6 30-min infusion [Tile/Abstract]

#7 randomized controlled trials [Text Word]

#8 # 1 and #2

#9 #3 or #4 or #5 or #6 or #7

#10 #8 and #9
